# Immunologic findings precede rapid lupus flare after transient steroid therapy

**DOI:** 10.1038/s41598-019-45135-w

**Published:** 2019-06-13

**Authors:** Rufei Lu, Joel M. Guthridge, Hua Chen, Rebecka L. Bourn, Stan Kamp, Melissa E. Munroe, Susan R. Macwana, Krista Bean, Sudhakar Sridharan, Joan T. Merrill, Judith A. James

**Affiliations:** 1 Arthritis and Clinical Immunology, Oklahoma Medical Research Foundation, Oklahoma City, OK 73104 USA; 2 Departments of Pathology and Medicine, University of Oklahoma Health Sciences Center, Oklahoma City, OK 73104 USA; 3 Pharmaceutical Product Development, Inc, Rockville, MD 20850 USA

**Keywords:** B cells, Monocytes and macrophages, Prognostic markers, Immunopathogenesis, Systemic lupus erythematosus

## Abstract

Systemic lupus erythematosus (SLE) flares elicit progressive organ damage, leading to disability and early mortality. This study evaluated clinical and immunologic factors associated with impending flare in the Biomarkers of Lupus Disease study. Autoantibodies and 32 soluble mediators were measured by multiplex assays, immune pathway activation by gene expression module scores, and immune cell subset frequencies and activation states by flow cytometry. After providing baseline samples, participants received transient steroids to suppress disease and were followed until flare. Flare occurred early (within 60 days of baseline) in 21 participants and late (90–165 days) in 13. At baseline, compared to the late flare group, the early flare group had differential gene expression in monocyte, T cell, interferon, and inflammation modules, as well as significantly higher frequencies of activated (aCD11b^+^) neutrophils and monocytes, and activated (CD86^hi^) naïve B cells. Random forest models showed three subgroups of early flare patients, distinguished by greater baseline frequencies of aCD11b^+^ monocytes, or CD86^hi^ naïve B cells, or both. Increases in these cell populations were the most accurate biomarkers for early flare in this study. These results suggest that SLE flares may arise from an overlapping spectrum of lymphoid and myeloid mechanisms in different patients.

## Introduction

Systemic lupus erythematosus (SLE) is a chronic, debilitating autoimmune disease that causes irreversible organ damage, contributing to diminished quality of life and early mortality^1,2^. Most SLE patients experience periods of relatively quiescent disease punctuated with periods of increased clinical activity^3^. Because disease flares and the major immunosuppressants used to subdue disease activity can both cause irreparable damage^1^, the frequency and severity of flares are important prognostic indicators for long term SLE outcomes^4–6^. In patients receiving standard-of-care treatments, rates of flare range from 0.24 to 1.8 flares per person-year^5–7^. In the phase III belimumab trials, 19.4% of patients who took steroids at baseline in addition to other standard treatments developed a flare over a 1-year period^7^.

Current clinical and laboratory instruments for forecasting disease flares have some utility but remain inadequate. Low complement C3 levels and rising anti-dsDNA have been useful as predictors of flare in the subset of patients with serologically active disease^8–15^. Cell-bound complement activation products have been correlated with SLE disease activity in longitudinal studies of patients with active disease and elevated complement activity at baseline^16^, but not all SLE patients have elevated cell-bound complement activation products^17^. Another proposed flare predictor, elevated frequency of CD27^hi^ plasma cells, was later shown to have a stronger association with infection than with disease flares^9^.

Currently available markers do not always predict impending flare^18–22^. If the timing of flares can be anticipated, it may be possible to optimize treatment to prevent flares while also sparing patients from unnecessary toxic treatments. Thus, predicting disease flares could reduce morbidity and improve early mortality rates in SLE patients. Being able to identify SLE patients with an increased risk of imminent flare would also help in the design of discriminatory clinical trials. Molecular mechanisms of disease flare are also largely unknown, and identification of such mechanisms may be important for therapeutic selection in SLE, as well as development of novel targeted therapies. Therefore, the goal of this study was to identify potential mechanisms of rapid flare after disease suppression through characterization of baseline soluble mediators, immune cell phenotypes, and gene expression patterns.

## Results

### Characteristics of patients with early or late flare

**Figure 1:**
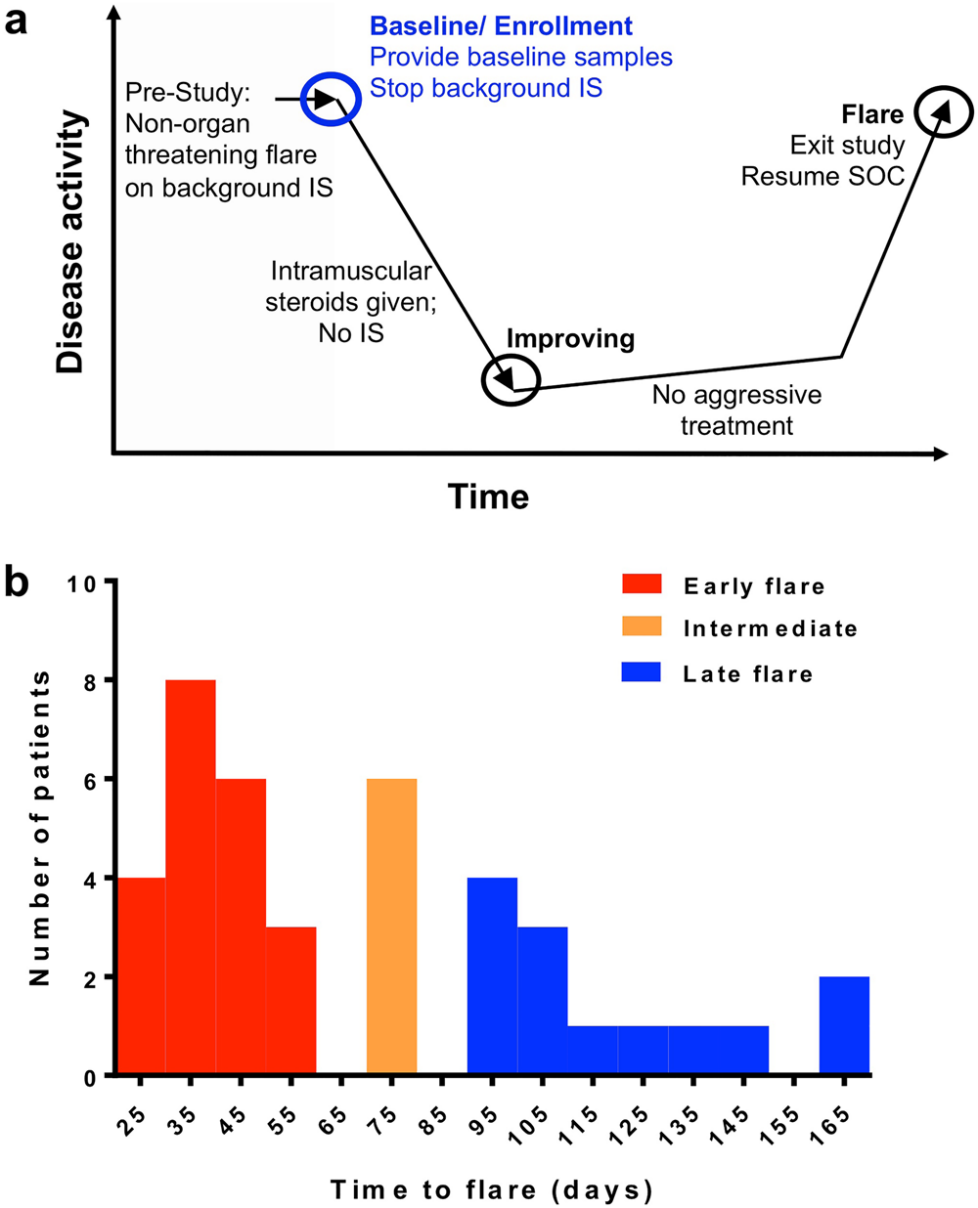
Identification of patients with early or late flare after steroid-induced disease suppression. (**a**) Samples and data from this study were obtained from the previously published Biomarkers of Lupus Disease (BOLD) clinical study^23^. (**b**) Patients were assigned to three groups based on time to flare during the BOLD study: early flare (<60 days; red; n = 21), intermediate flare (between 60 and 90 days; orange; n = 6), and late flare (90–165 days; blue; n = 13). The current study compared the early flare group vs. the late flare group. IS: immunosuppressant therapy; SOC: standard of care.

All evaluated patients had active, but not organ threatening, SLE at baseline and clinically significant improvement after receiving one to four bolus depomedrol injections (160 mg each; ≤640 mg total). Background immunosuppressants were withdrawn and patients serially followed until worsening of disease activity associated with clinician’s intention to treat (flare visit) as described^23^. Of 41 participants, 40 (97.6%) had a flare within 6 months. Of these, 21 had a flare within 60 days (early flare; median time-to-flare of 38 days), and 13 had a flare ≥90 days from baseline (late flare; median time-to-flare of 109 days) (Fig. 1, Table 1). Early flare was more common in African-American participants than others. Other demographic characteristics were similar between the early and late flare groups, as were the ACR criteria; baseline immunosuppressant usage (Table 1); baseline, maximally suppressed and flare disease activity (Supplementary Fig. S1); clinical lab values (Supplementary Table S1); and prevalence and levels of antinuclear autoantibodies measured at baseline (Supplementary Table S2). The early and late flare groups showed no significant differences in the number of steroid injections required to achieve clinical improvement (early flare: median 2, range 2–6; late flare: median 3, range 2–4; p = 0.126).

**Table 1:** Demographics and baseline clinical characteristics of study cohort.

	Early Flare (n = 21)	Late Flare (n = 13)	Odds Ratio (95% CI)	P value^A^
**Gender**				
Female, n (%)	21 (100%)	11 (85%)	9.3 (0.4, 212)	0.06
**Ethnicity**				
European American, n (%)	12 (57%)	7 (53.9%)	8.6 (0.8, 89)	0.04^B^ 0.16 ^C^
African American, n (%)	8 (38%)	1 (7.7%)	40 (2, 795)	0.005^D^
American Indian, n (%)	1 (5%)	3 (23.1%)	NC	
Hispanic, n (%)	0 (0%)	5 (15.3%)	NC	
Age, Median (Range)	43 (25–58)	32 (22–62)		0.35
ACR Criteria, Median (Range)	5 (4–9)	6 (4–8)		0.51
Malar Rash, n (%)	9 (43%)	9 (69%)	0.33 (0.08, 1.4)	0.13
Discoid Rash, n (%)	8 (38%)	5 (38%)	0.98 (0.24, 4.1)	0.84
Photosensitivity, n (%)	14 (67%)	11 (85%)	0.36 (0.6, 2.1)	0.25
Oral Ulcer, n (%)	16 (76%)	9 (69%)	1.4 (0.4, 6.7)	0.65
Arthritis, n (%)	20 (95%)	13 (100%)	0.51 (0.02, 13)	0.42
Serositis, n (%)	9 (43%)	2 (15%)	4.1 (0.73, 23)	0.10
Renal Disorder, n (%)	4 (19%)	0 (0%)	6.9 (0.34, 141)	0.09
Neurologic Disorder, n (%)	2 (9%)	2 (15%)	0.58 (0.07, 4.7)	0.61
Hematologic Disorder, n (%)	5 (24%)	5 (38%)	0.5 (0.11, 2.2)	0.36
Immunologic Disorder, n (%)	13 (62%)	9 (69%)	0.72 (0.17, 3.1)	0.66
ANA, n (%)	21 (100%)	13 (100%)	NC	NC
**Medication**				
Azathioprine, n (%)	5 (24%)	5 (38%)		0.60
Hydroxychloroquine, n (%)	15 (71%)	10 (77%)		1.0
Methotrexate, n (%)	5 (24%)	2 (15%)		0.68
Mycophenolate mofetil, n (%)	3 (14%)	2 (15%)		1.0
Prednisone, n (%)	2 (10%)	2 (15%)		1.0
Days to flare, Median (Range)	38 (22–56)	109 (91–168)		<0.001

^A^Calculated by chi-squared test or Fisher test. ^B^Compared to all non-European American patients. ^C^Compared to African-American patients. ^D^Compared to all non-African-American patients. NC: Not calculable.

### Distinct molecular signatures in early and late flare groups at baseline

**Figure 2:**
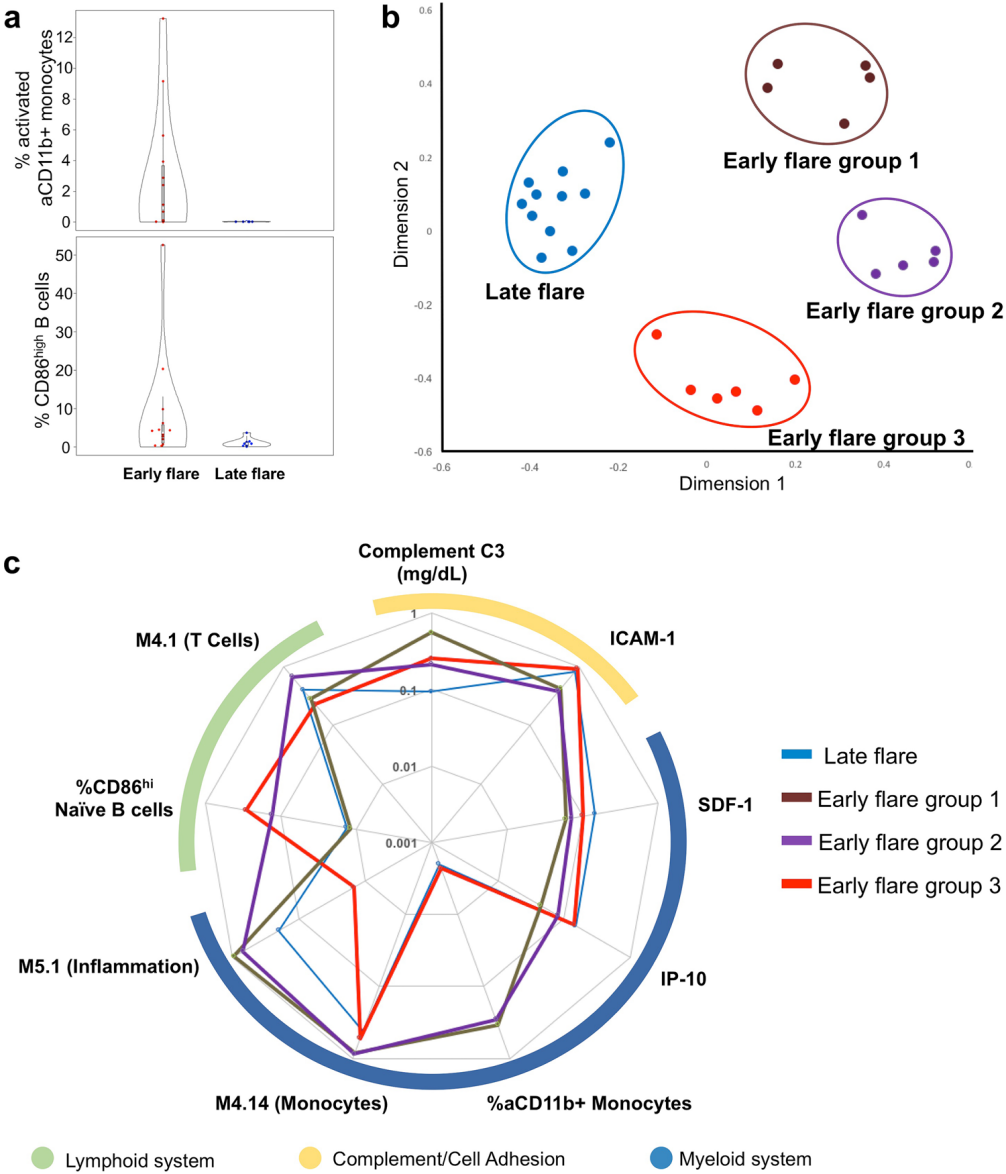
Transcriptional modules at baseline in SLE patients with early or late flare. (**a**,**b**) Activation of transcriptional modules was determined at baseline and compared between SLE patients with early flare (**a**; n = 21) or late flare (**b**; n = 13) versus healthy controls. Each box marked with a colored square represents a module, and the color of the square indicates the primary function of the module, as shown at the bottom of the figure. The size of each circle represents the absolute value of the module score. The color represents an increase (red circles; positive scores) or decrease (blue circles; negative scores) in the pathway, in patients compared to controls, as shown at right. P values were determined by non-parametric test. (**c**) The radar plot summarizes differences between the module scores in the early vs. late flare groups.

To identify molecular patterns that preceded early flares, we analyzed baseline samples for transcriptional modules that were previously associated with immune modulation^24,25^. The early flare group exhibited an elevated interferon signature (M3.4, M5.12), a slightly elevated inflammation signature (M4.6, M5.7; Fig. 2), and a suppressed T cell signature (M4.1, M4.15). The late flare group exhibited a suppressed inflammation signature (M3.2, M4.2, M4.6, M4.13, M5.1, M5.7), a slightly decreased monocyte signature (M4.14), and a slightly elevated T cell signature (Fig. 2). These differences in molecular pathway signatures suggest that the timing of SLE flares may be influenced by functional characteristics in the myeloid and lymphoid compartments.

### Baseline plasma levels of IFN-γ, TNFRII and TNFRI in patients with early or late flare

To further explore immune pathways that may influence time to flare, we compared 32 cytokines, chemokines, adhesion molecules, and soluble tumor necrosis factor receptors (TNFR) at baseline. The early flare group had lower levels of IFN-γ compared to the late flare group, along with slightly lower concentrations of TNFRI and TNFRII (Supplementary Table S3).

### Frequencies of activated naïve B cells (CD86^hi^), neutrophils (activated CD11b^+^), and monocytes (activated CD11b^+^) preceding early or late flare

**Figure 3:**
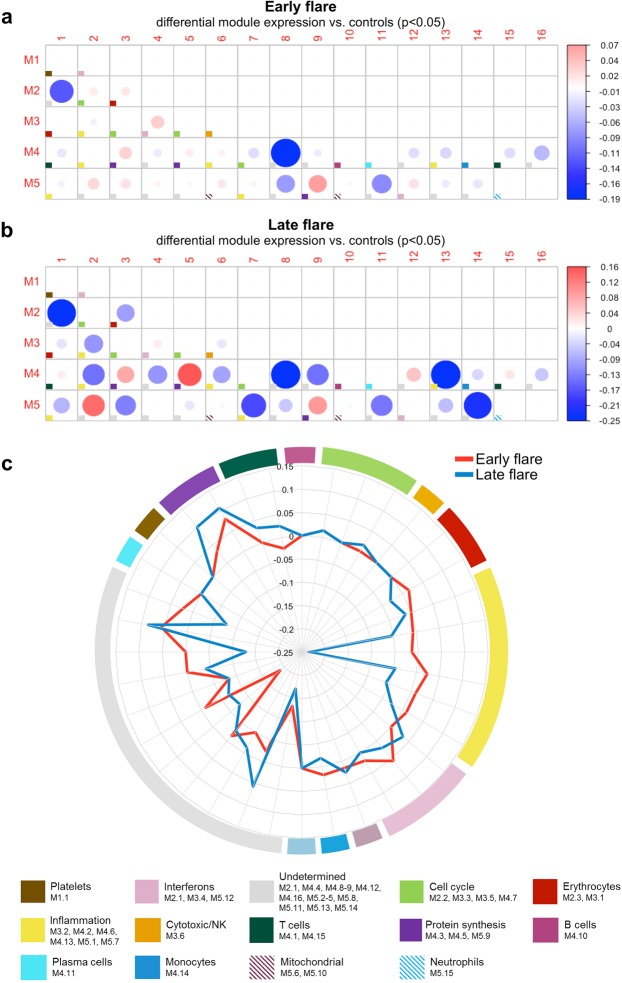
Frequencies of aCD11b+ monocytes and CD86^hi^ naïve B cells distinguish SLE patients with early or late flare in random forest modeling. (**a**) Baseline frequencies of activated CD11b positive (aCD11b+) monocytes (top) and CD86^high^ B cells (bottom) were quantified by flow cytometry in patients with early flare (n = 21) or late flare (n = 13) after steroid-induced disease suppression. For both comparisons, p < 0.05 by Mann-Whitney U test. (**b**) Random forest modeling with cellular, clinical, cytokine, and transcriptional panels identified late flare patients and three subgroups of early flare patients. The random forest model proximity matrix is shown as a multi-dimensionally reduced plot, where each point represents a patient, and the distance between two points represents dissimilarity between patients. (**c**) The variables included in the final random forest model for each independent panel (cellular, clinical, cytokine, and genetic module) are shown in a radial plot. Variables are grouped according to their involvement in the lymphoid, complement/cell adhesion, or myeloid system. Lines represent normalized values for the late flare group and the three early flare subgroups, as indicated.

Alterations in cellular profiles have been associated with SLE disease activity and immune dysregulation. Therefore, cellular profiles were assessed by flow cytometry (Supplementary Tables S4, S5). Expression of activated CD11b (aCD11b), determined by a conformation-specific antibody, was used as an indicator of neutrophil and monocyte activation. Patients with an early flare had significantly higher frequencies of activated neutrophils and monocytes compared to patients with a late flare (Fig. 3, Supplementary Table S5). In addition to the altered myeloid compartment, the early flare group had a significantly elevated frequency of activated naïve B cells (CD86^hi^) compared to the late flare group (Fig. 3, Supplementary Table S5). These results suggest that early flares were preceded by dysregulation in activation of the lymphoid and/or myeloid compartments.

### Three subsets of early flare patients differentiated by elevated frequencies of activated monocytes, activated naïve B cells, or both

Because of the complexity of immune dysregulation in SLE, characterizing pathways related to imminent clinical flares requires a multifactorial model capable of handling various data types (e.g. categorical and continuous) and detecting interactions between variables. The random forest classification algorithm distills large, multifactorial datasets down to the handful of variables that are most informative as independent classifiers. Therefore, to better understand the immune pathways that preceded early flares, we performed random forest modeling with each panel of variables (clinical, cellular phenotypes, expression modules, and soluble mediators) to develop four independent models for early vs. late flare (Table 2).

**Table 2:** Models for early vs. late SLE flare after steroid-induced disease suppression, based on random forest.

Panel	Variables in final model	Sensitivity (95% CI)	Specificity (95% CI)	PPV (95% CI)	NPV (95% CI)	Overall error (% ±SD)
Cellular	%aCD11b + Monocytes, %CD86^hi^ Naïve B	0.92 (0.64, 1.00)	0.79 (0.44, 0.97)	0.85 (0.56, 0.98)	0.89 (0.52, 1.00)	13.56 ± 1.42
Clinical	Complement C3	0.93 (0.68, 1.00)	0.64 (0.35, 0.87)	0.74 (0.49, 0.91)	0.90 (0.55, 1.00)	20.69 ± 0.01
Cytokine	SDF1, ICAM-1, IP-10	0.72 (0.51, 0.88)	0.75 (0.35, 0.97)	0.90 (0.68, 0.99)	0.46 (0.19, 0.75)	27.05 ± 0.60
Expression modules	M5.1 (Inflammation), M4.14 (Monocytes), M4.1 (T Cells)	0.71 (0.42, 0.92)	0.57 (0.18, 0.90)	0.77 (0.46, 0.95)	0.50 (0.16, 0.84)	33.33 ± 0.01
Total	%CD11b + Monocytes, %CD86^hi^ Naïve B	1.00 (0.29, 1.00)	0.61 (0.15, 0.95)	0.69 (0.24, 0.96)	1.00 (0.19, 1.00)	20.89 ± 3.65

PPV: positive predictive value, NPV: negative predictive value.

Of these baseline models for early flare, the model based on cellular phenotypes (n = 30) was the most reliable (86.4 ± 1.4% accuracy). In this model, higher frequencies of activated (aCD11b^+^) monocytes (>0.2%), or activated (CD86^hi^) naïve B cells (>2.3%), or both were the strongest markers of early flare (Fig. 3, Table 2). This model revealed three subsets of patients within the early flare group: (1) patients with high frequencies of aCD11b^+^ monocytes and normal frequencies of CD86^hi^ naïve B cells, (2) patients with high frequencies of CD86^hi^ naïve B cells and normal frequencies of aCD11b^+^ monocytes, and (3) patients with increased frequencies of both cell populations (Fig. 3). Although molecularly heterogeneous, clinical features were similar across the three subsets of patients with early flare (Supplementary Table S6). Within the subgroups of early flare patients, increases in aCD11b^+^ monocytes and CD86^hi^ naïve B cells corresponded to higher scores in the M4.10 B cell and M4.14 monocyte correlated gene expression modules, respectively (Figs 3, S2).

The clinical panel produced the second most accurate independent model (79.31 ± 0.01% accuracy), based on complement C3 levels (Table 2). The model based on soluble mediators reached 72.95 ± 0.60% accuracy, based on levels of the CD11b receptor ICAM-1 and the IFN-γ-associated mediator IP-10 (Table 2, Supplementary Fig. S2). Levels of ICAM-1 inversely correlated with the frequency of activated aCD11b^+^ monocytes (Supplementary Fig. S2). The model based on co-expression module scores reached 66.67% ± 0.01% accuracy, based on the M5.1 inflammation, M4.14 monocyte, and M4.1 T cell module scores.

Finally, in a comprehensive prediction model using only the top biomarkers from each panel, aCD11b^+^ monocytes and CD86^hi^ naïve B cells frequencies were the strongest predictors of early flare. The comprehensive model outperformed the models based on gene expression modules or soluble mediators alone (88.67 ± 2.69% accuracy; Table 2). However, the comprehensive model was limited by missing values, and underperformed against the cellular phenotypes model. Therefore, elevated frequencies of aCD11b^+^ monocytes, CD86^hi^ naïve B cells, or both were the most reliable and accurate biomarkers for early flare in this study. These results indicate that SLE flares may follow dysregulation of lymphoid, myeloid, or both pathways.

## Discussion

Although enhanced diagnostic tools and increased use of immunosuppressive therapies have reduced SLE-related mortality in the past fifty years, organ damage caused by disease flares and aggressive treatment continues to impede quality of life and long-term survival^1,26–28^. The current study has identified baseline molecular and cellular signatures that distinguish between patients with early and late flare after steroid-induced disease suppression, and reveal multiple pathways preceding flare in these patients.

As expected in a heterogenous disease such as SLE, no single biomarker distinguished all patients with early flare. In the most accurate model, frequencies of activated (aCD11b^+^) monocytes, or CD86^hi^ naïve (CD19^+^/CD27^−^) B cells, or both were sufficient to distinguish patients who had an early flare from those with delayed flare. These variables also defined three distinct subsets of patients with early flare, suggesting that activity of myeloid cells and lymphocytes could contribute, either separately or synergistically, to imminent flares in different subsets of SLE patients. Therefore, an overlapping spectrum of immune dysregulation may lead to SLE flares in different patients. The identification of biomarkers associated with early flare in this study has potential implications for disease monitoring and treatment optimization. This warrants confirmation in a larger population, with detailed immunological analyses of samples collected before, during, and after steroid treatment.

In one patient subset, early flares were preceded by high frequencies of activated (aCD11b^+^) monocytes, elevated monocyte module scores, and decreased plasma concentrations of ICAM-1, a CD11b receptor. This is consistent with other data supporting an important role for myeloid cells in SLE pathophysiology^29,30^ and a specific association of CD11b with disease activity^31^. Functional variants in ITGAM, the gene encoding CD11b, strongly associate with SLE in case-control studies^32^ and with lupus nephritis, discoid rash, and immunologic manifestations in case-only analyses^33^. In murine models, activated renal macrophages are a prominent feature of lupus nephritis, corroborating an association between macrophage activation and disease severity^34^. In addition, CD11b is a component of complement receptor 3, which recognizes iC3b-coated immune complexes and initiates stimulatory pathways in monocytes and neutrophils^35–38^.

In another subset of early flare patients, elevated frequencies of activated CD86^hi^/CD19^+^/CD27^−^ naïve B cells and increased B cell module scores suggest that flares in some individuals are preceded by perturbed B cell homeostasis. Indeed, abnormal expansion of activated B cells in SLE patients has been correlated with disease activity^39,40^. Finally, the third subset of early flare patients exhibited increases in both aCD11b^+^ monocytes and CD86^hi^ B cells at baseline, suggesting that these pathways may act in concert to elicit flares in some patients.

The interferon modules (M1.2, M3.4, and M5.12) were elevated in all groups of SLE patients in this study compared to healthy controls. However, patients with early flare showed a further increase compared to patients with late flare, suggesting a continuum of interferon dysregulation. In addition, different interferon types induce different immune responses and may affect the efficacy of glucocorticoids^25,41–43^. In this study, patients with late flare had slightly higher levels of IFN-γ than patients with early flare. IFN-γ is associated with regulatory pathways in addition to its role in monocyte and B cell activation, and it is possible that production of IFN-γ by regulatory cells would suppress flares in some patients. Alternatively, a slight increase in IFN-γ may arise from occult infection, Th1 cells, or other sources. Therefore, additional studies are needed to determine the source of IFN-γ and to confirm its influence on lupus flares.

In conclusion, this study identified immune variables that distinguish SLE patients with rapid flare after transient corticosteroid-induced disease suppression, suggesting that flares may arise through immune pathways involving activation of naïve B cells, monocytes or both. The ability to identify patients at risk of impending flare could optimize the timing of disease suppression therapy and contribute to more effective and efficient clinical trial designs.

## Methods

### Patients and samples

This study was approved by the Oklahoma Medical Research Foundation Institutional Review Board and conducted in accordance with the Helsinki Declaration. Written informed consent was obtained prior to study-specific procedures.

These experiments used data and samples obtained at the baseline visit of the Biomarkers of Lupus Disease (BOLD) study (NCT00987831), which recruited SLE patients with moderate to severe, but non-organ threatening disease^23^. Upon enrollment (baseline), background immunosuppressants were withdrawn, with the option to continue hydroxychloroquine and up to 10 mg/day prednisone or equivalent steroid. Patients received transient steroid injection(s) until disease activity was reduced (improving visit), and were closely followed until the first signs of a flare, at which time they were immediately treated (Fig. 1, Supplementary Methods).

Clinical evaluations included the SLE disease activity index (SLEDAI), British Isles Lupus Assessment Group (BILAG) 2004 Index, physician global assessments (PGA)^44–47^, complete blood counts (CBC) with differential, blood chemistries, urine analysis, and clinical-serologic markers of SLE (Supplementary Table S1). Baseline peripheral blood specimens, peripheral blood mononuclear cells (PBMCs), and PAXgene tubes were collected as described (Supplementary Methods and^23^).

### Modular analysis of gene expression molecular signatures

mRNA was isolated from whole blood baseline samples of 21 SLE patients and 15 healthy controls from the BOLD study. mRNA expression in globin-depleted peripheral blood was measured with the HumanHT-12 v4 Expression BeadChip (Illumina, San Diego, California). Quality control was performed with Illumina GenomeStudio Version 2011.1 (Illumina, San Diego, California) per manufacturer protocol.

Gene expression data were analyzed using R version 3.3.2. Background-subtracted expression data were log2 transformed and normalized with the rankinvariant method using the lumiR package^48^. System-based modular analysis at the group level and the individual level was performed using second generation modular frameworks as described^24,25^. Individual-level based modular scores were used for subsequent Random Forest prediction models.

### Soluble mediator and autoantibody assessments

Antinuclear antibodies (dsDNA, chromatin, ribosomal P, 52 and 60 kD Ro/SS-A, La/SS-B, Sm, Sm/RNP, RNP A and 68 kD RNP, Scl-70, Jo-1, and centromere B) were measured using the autoimmune disease panel on the Bioplex® 2200 (Bio-Rad, Hercules, California) as described^49–51^.

Plasma levels of B lymphocyte stimulator (BLyS; R&D Systems, Minneapolis, MN) and a proliferation-inducing ligand (APRIL; eBioscience/Affymetrix, San Diego, CA) were determined by enzyme-linked immunosorbent assays (ELISA).

Additional analytes including innate and adaptive cytokines, chemokines, and soluble tumor necrosis factor receptor (TNFR) superfamily members were assessed using a custom multiplex panel (ProcartaPlex, Thermo Fisher Scientific/Invitrogen) on the Bioplex 200 Luminex xMAP plate reader (Bio-Rad Technologies, Hercules, CA) as described^52,53^. Well-specific validity was assessed by AssayCheX™ QC microspheres (Radix BioSolutions, Georgetown, TX, USA), and a standard control serum was included on each plate (Cellgro human AB serum, Cat#2931949, L/N#M1016). Data were acquired on the BioPlex 200 array system (Bio-Rad Technologies, Hercules, CA), with a lower boundary of 100 beads per sample/analyte. Mean inter-assay coefficient of variance (CV) using healthy control serum was 10.5%, similar to other multiplexed cytokine bead-based assays^54^.

Limit of blank, limit of detection, and limit of quantification were determined using the method of the College of American Pathologists and the Clinical and Laboratory Standards Institute^55–58^^.^ The quality control algorithm is detailed in Supplementary Fig. S3. Analytes with >60% undetectable rate were excluded from subsequent analyses. For the 32 analytes passing quality control (Supplementary Table S3), concentrations were interpolated from 5-parameter logistic nonlinear regression standard curves, or assigned a value of 0 if a sample was below the limit of detection.

### Immune cell profiling

Immune cells in whole blood were assess by flow cytometry with antibodies for T cell, B cell, and monocyte profiling (Supplementary Methods and Supplementary Table S4). Activated neutrophils and monocytes were identified by expression of anti-activated CD11b (CBRM1/5), which specifically recognizes the CD11b epitope exposed by activation-induced conformational change, above the positive cutoff determined by an isotype control (Supplementary Fig. S4). Debris and doublets were excluded using forward scatter (FSC) and side scatter (SSC). Lymphocytes, monocytes, and granulocytes were roughly separated based on FSC and SSC (Supplementary Fig. S4). Monocytes were further identified by total CD11b expression, and neutrophils were distinguished by CD16 expression. A histogram of fluorescence intensity was used to assess each surface marker. When the histogram showed uni-modal distribution, the median fluorescence intensity was used for further analysis, whereas the 95^th^ percentile was used to define the cut-offs for high expressing populations in multimodal cellular distributions. All flow cytometric results were analyzed by one individual and checked by a second who are both trained in flow cytometry analysis to ensure consistency in data acquisition and analysis.

### Statistical analyses

Non-parametric analysis of numerical variables was performed using GraphPad Prism 6.0 (La Jolla, CA). Categorical variables were analyzed using chi-square test, and odds ratios with 95% confidence interval were calculated. False discovery rate (FDR) was used to adjust for multiple testing with the fdr tool R package. A correlation matrix of variables retained after quality control was calculated using R version 3.3.2 and plotted with a p-value filter using corrplot R packages.

Random forest classification was performed in R version 3.3.2 using the randomForest package by Breiman and Cutler, with mtry (number of splitting variables tried at each node) = $\sqrt{no.variables}$ and ntree (number of decision trees) = 2000^59^. This algorithm generates multiple recursive decision trees based on a randomly selected two-thirds of the samples, then uses the ensemble of decision trees to predict outcomes in the remaining one-third of samples to calculate an internal error, as described^60^. For the stepwise algorithm, 25 forests were initially used to generate average variable importance with standard deviation based on unscaled accuracy reduction after variable permutation. Noisy variables were excluded based on variable importance, as described^61^^.^ Briefly, variables were added iteratively in order of importance (from most to least important) to a nested prediction model. Only variables that significantly reduced the prediction error were retained in the final model. Sensitivity, specificity, positive predictive value, and negative predictive value were calculated based on the averaged confusion matrix of 50 forests generated using the best model.

## Supplementary information

Supplement

## Data Availability

The datasets generated during and/or analyzed during the current study are available from the corresponding author upon reasonable request.
