# Author Correction: Immunologic findings precede rapid lupus flare after transient steroid therapy

**DOI:** 10.1038/s41598-019-54275-y

**Published:** 2019-11-20

**Authors:** Rufei Lu, Joel M. Guthridge, Hua Chen, Rebecka L. Bourn, Stan Kamp, Melissa E. Munroe, Susan R. Macwana, Krista Bean, Sudhakar Sridharan, Joan T. Merrill, Judith A. James

**Affiliations:** 1 Arthritis and Clinical Immunology, Oklahoma Medical Research Foundation, Oklahoma City, OK 73104 USA; 2 Departments of Pathology and Medicine, University of Oklahoma Health Sciences Center, Oklahoma City, OK 73104 USA; 3 Pharmaceutical Product Development, Inc, Rockville, MD 20850 USA

Correction to: *Scientific Reports* 10.1038/s41598-019-45135-w, published online 13 June 2019

This Article contains an error in the order of the Figures. Figure 2 was published as Figure 3, and Figure 3 was published as Figure 2. The Figure legends were published in the correct order. The correct Figures 2 and 3 appear below as Figures 1 and 2, along with their corresponding legends.

**Figure 1:**
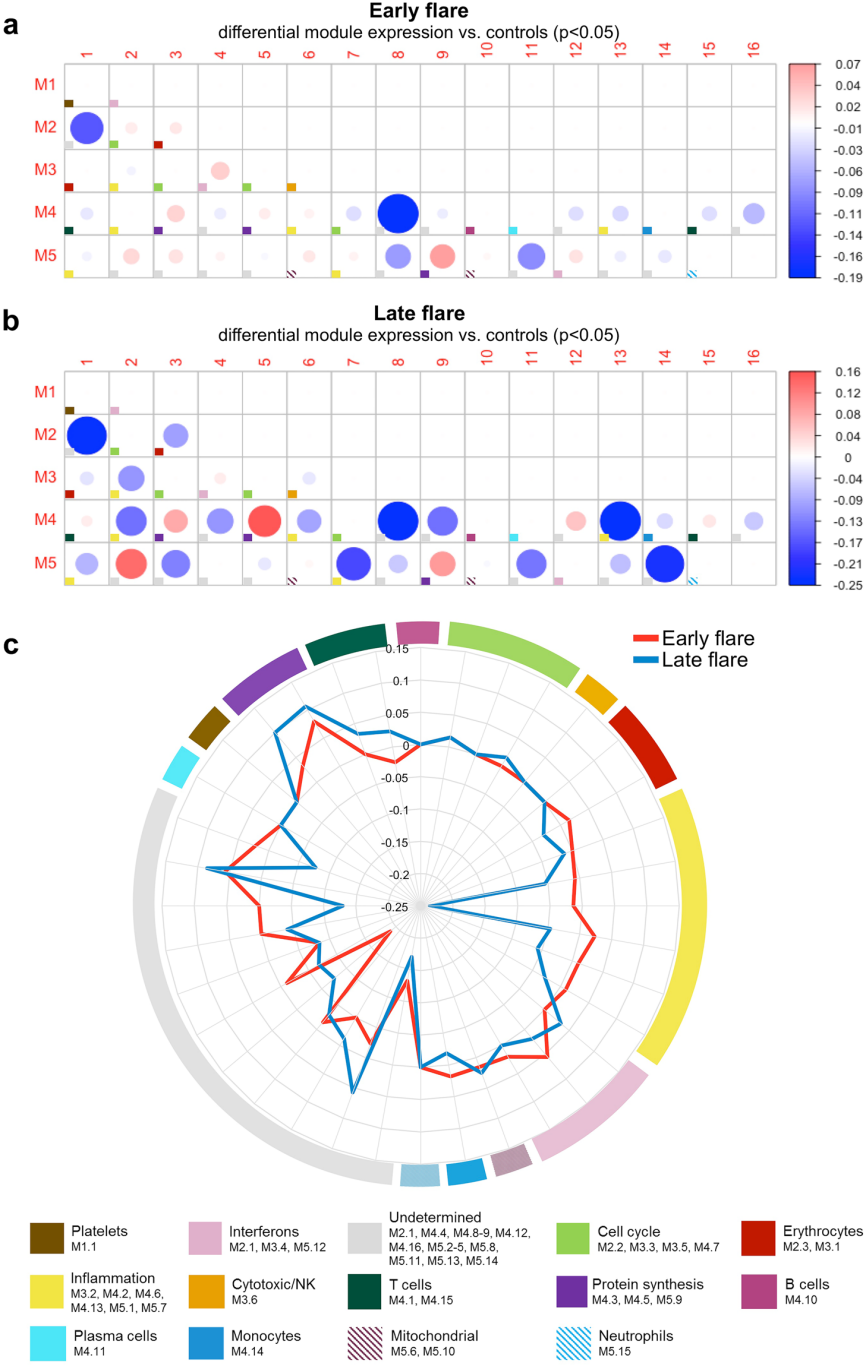
Transcriptional modules at baseline in SLE patients with early or late flare. (**a**,**b**) Activation of transcriptional modules was determined at baseline and compared between SLE patients with early flare (**a**; n = 21) or late flare (**b**; n = 13) versus healthy controls. Each box marked with a colored square represents a module, and the color of the square indicates the primary function of the module, as shown at the bottom of the figure. The size of each circle represents the absolute value of the module score. The color represents an increase (red circles; positive scores) or decrease (blue circles; negative scores) in the pathway, in patients compared to controls, as shown at right. P values were determined by non-parametric test. (**c**) The radar plot summarizes differences between the module scores in the early vs. late flare groups.

**Figure 2:**
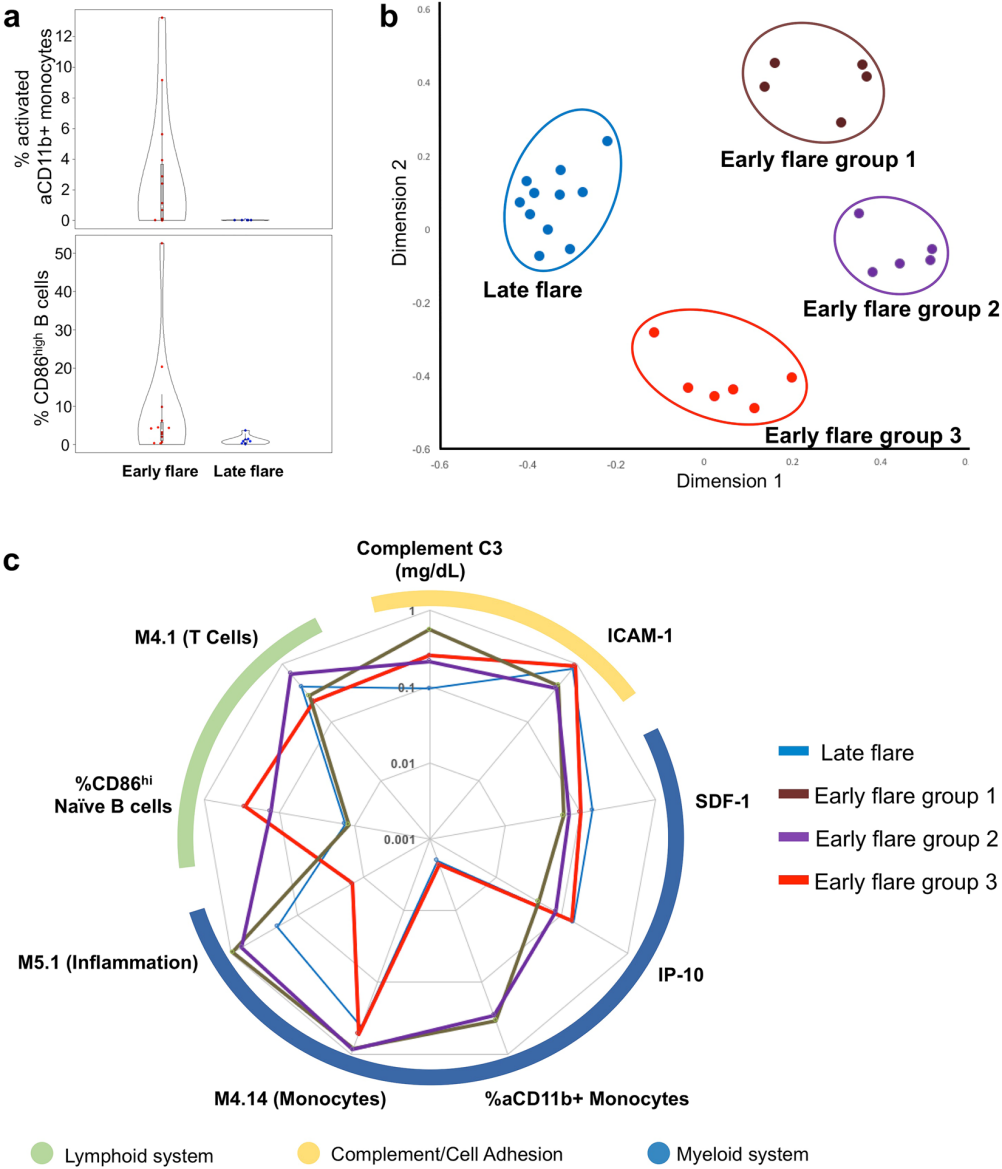
Frequencies of aCD11b+ monocytes and CD86^hi^ naïve B cells distinguish SLE patients with early or late flare in random forest modeling. (**a**) Baseline frequencies of activated CD11b positive (aCD11b+) monocytes (top) and CD86^high^ B cells (bottom) were quantified by flow cytometry in patients with early flare (n = 21) or late flare (n = 13) after steroid-induced disease suppression. For both comparisons, p < 0.05 by Mann-Whitney U test. (**b**) Random forest modeling with cellular, clinical, cytokine, and transcriptional panels identified late flare patients and three subgroups of early flare patients. The random forest model proximity matrix is shown as a multi-dimensionally reduced plot, where each point represents a patient, and the distance between two points represents dissimilarity between patients. (**c**) The variables included in the final random forest model for each independent panel (cellular, clinical, cytokine, and genetic module) are shown in a radial plot. Variables are grouped according to their involvement in the lymphoid, complement/cell adhesion, or myeloid system. Lines represent normalized values for the late flare group and the three early flare subgroups, as indicated.

