# Supplementary material for: Immunologic findings precede rapid lupus flare after transient steroid therapy
Source: Sci Rep. 2019 Jun 13;9:8590. doi: 10.1038/s41598-019-45135-w (PMC6565690; doi:10.1038/s41598-019-45135-w)
Supplement: Supplementary file 1 — Supplement [file 41598_2019_45135_MOESM1_ESM.docx]

**Supplementary Material**

**Immunologic findings precede rapid lupus flare after transient steroid therapy**

**Rufei Lu^1,2+^, Joel M. Guthridge^1,2+^, Hua Chen^1^, Rebecka L. Bourn^1^, Stan Kamp^1^, Melissa E. Munroe^1^, Susan R. Macwana^1^, Krista Bean^1^, Sudhakar Sridharan^3^, Joan T. Merrill^1^ and Judith A. James^1,2*^**

^1^ Arthritis and Clinical Immunology, Oklahoma Medical Research Foundation, Oklahoma City, OK, 73104, USA

^2^ Departments of Pathology and Medicine, University of Oklahoma Health Sciences Center, Oklahoma City, OK, 73104, USA

^3^ Pharmaceutical Product Development, Inc, Rockville, MD, 20850, USA

^+^Authors contributed equally to analysis and design of the study

*Corresponding Author: Judith A. James, MD, PhD, Arthritis and Clinical Immunology, Oklahoma Medical Research Foundation, 825 NE 13^th^ Street, Oklahoma City, OK 73104; Phone: (405) 271-4987; Fax: (405) 271-7063; email: [judith-james@omrf.org](mailto:jamesj@omrf.org)

**Supplementary Methods**

**Biomarkers of Lupus Disease (BOLD) Study Design**

The BOLD study has been described in detail (NCT00987831) ^1^. Briefly, all participants met ≥4 American College of Rheumatology (ACR) 1997 revised classification criteria^2,3^ and had SLEDAI scores ≥ 6 or BILAG scores of 1A (restricted to the mucocutaneous and musculoskeletal systems) or ≥2B at the time of enrollment. Upon enrollment (baseline visit), patients withdrew background immunosuppressants and received transient steroid injection(s) until disease activity was reduced (improving visit). Only patients who improved within two weeks of baseline were allowed to continue in this protocol. Patients were then followed until clinical disease flare occurred (flare visit), as shown in Fig. 1 in the main text. Disease activity was measured by SLE disease activity index (SLEDAI), British Isles Lupus Assessment Group (BILAG) 2004, and physician global assessment (PGA).^2,4-6^ Flares were defined as at least one grade worsening by BILAG or four point increase in SLEDAI, compared to the improving visit, coupled with clinician’s opinion of significant worsening and intention to treat. Upon experiencing a disease flare, patients were immediately treated and exited the study. All end of study flares resolved within six weeks.

**Patients and samples**

Blood was collected from patients and controls in BD Vacutainer ACD tubes, BD Vacutainer Serum tubes, and PAXgene tubes. Sera and plasma were distributed into aliquots of up to 1 mL and stored at -80 ^O^C. T cells, B cells, and neutrophils were isolated from whole blood (Dynabeads CD2, Dynabeads CD19 Pan B, and Dynabeads CD15, respectively; ThermoFisher Scientific, Waltham, MA), suspended in a cryopreservative buffer, and stored at -80 ^O^C. For RNA, blood was drawn into 2.5 mL PAXgene tubes (ref#762165, PreAnalytiX, Switzerland), immediately inverted multiple times, and then stored upright for a minimum of two hours at room temperature, not exceeding 72 hours. Tubes were then frozen upright and stored at -80 ^O^C.

**Immune cell profiling**

For immune cell profiling, within 8 hours of sample draw, whole blood was aliquoted into BD FACS tubes (BD Biosciences). Samples were mixed with antibodies for flow cytometry, in freshly prepared 1X FACS lysing solution, and incubated for 30 minutes. Antibodies included panels for T cell, B cell, and monocyte profiling (Supplementary Table 3) as well as anti-activated CD11b (CBRM1/5), which specifically recognizes the CD11b epitope exposed by activation-induced conformational change. After incubation, samples were washed twice with freshly prepared wash buffer (1mL; 1X PBS, 0.1% azide) and 1% paraformaldehyde (300 µL), then resuspended in wash buffer.

Flow cytometry data were collected on a BD LSR II cytometer and analyzed using FlowJo (TreeStar Inc., Ashland, OR). The same compensation controls were used for all samples, with the same voltage gains for each channel to minimize technical variability during data acquisition.


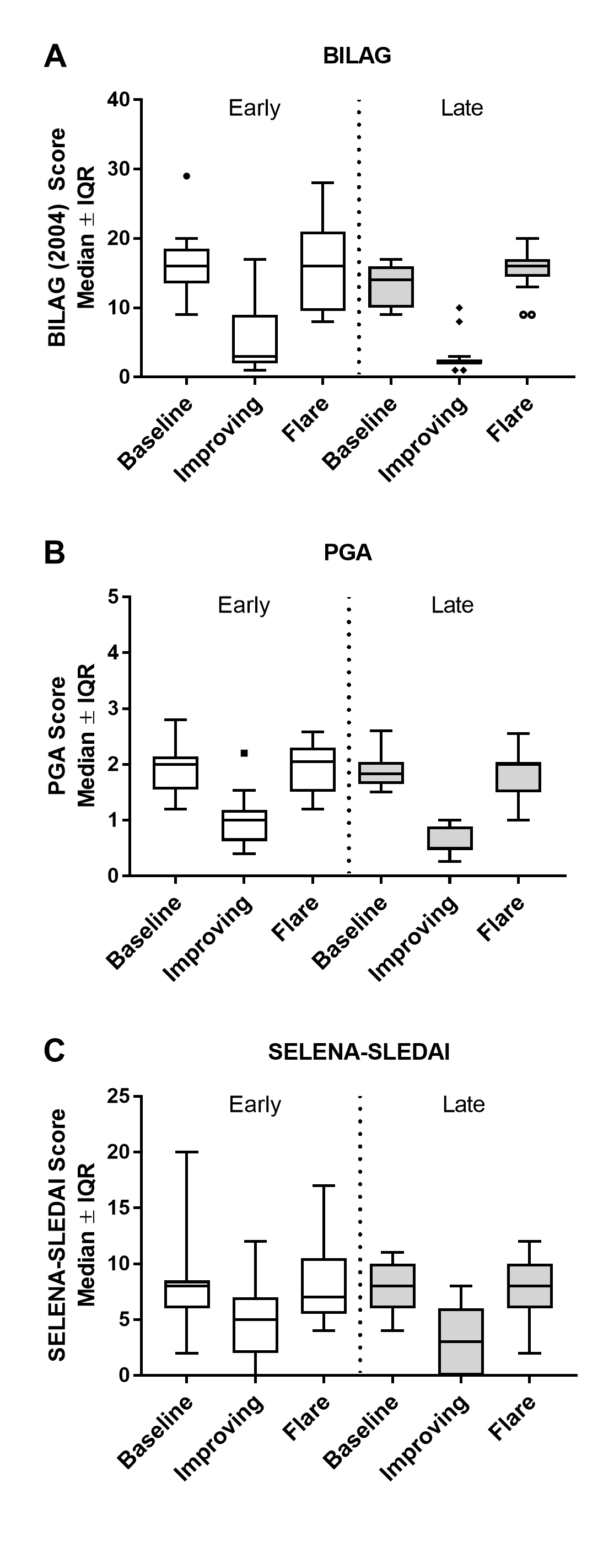


**Supplementary Figure S1**. Disease activity changes during the course of study were assessed by (**A**) 2004 BILAG, (**B**) Physician Global Assessment (PGA), and (**C**) SELENA-SLEDAI. Results are shown as a Tukey plot, where boxes indicate the median and interquartile range (IQR), whiskers indicate data within 1.5 IQR of the 25^th^ percentile and 50^th^ percentile, and dots show datapoints outside this range. The improving PGA was slightly higher in the early flare group than in the late flare group (p=0.0044) by Mann-Whitney test. All other comparisons between Early and Late flare were not significant (p>0.05).

**
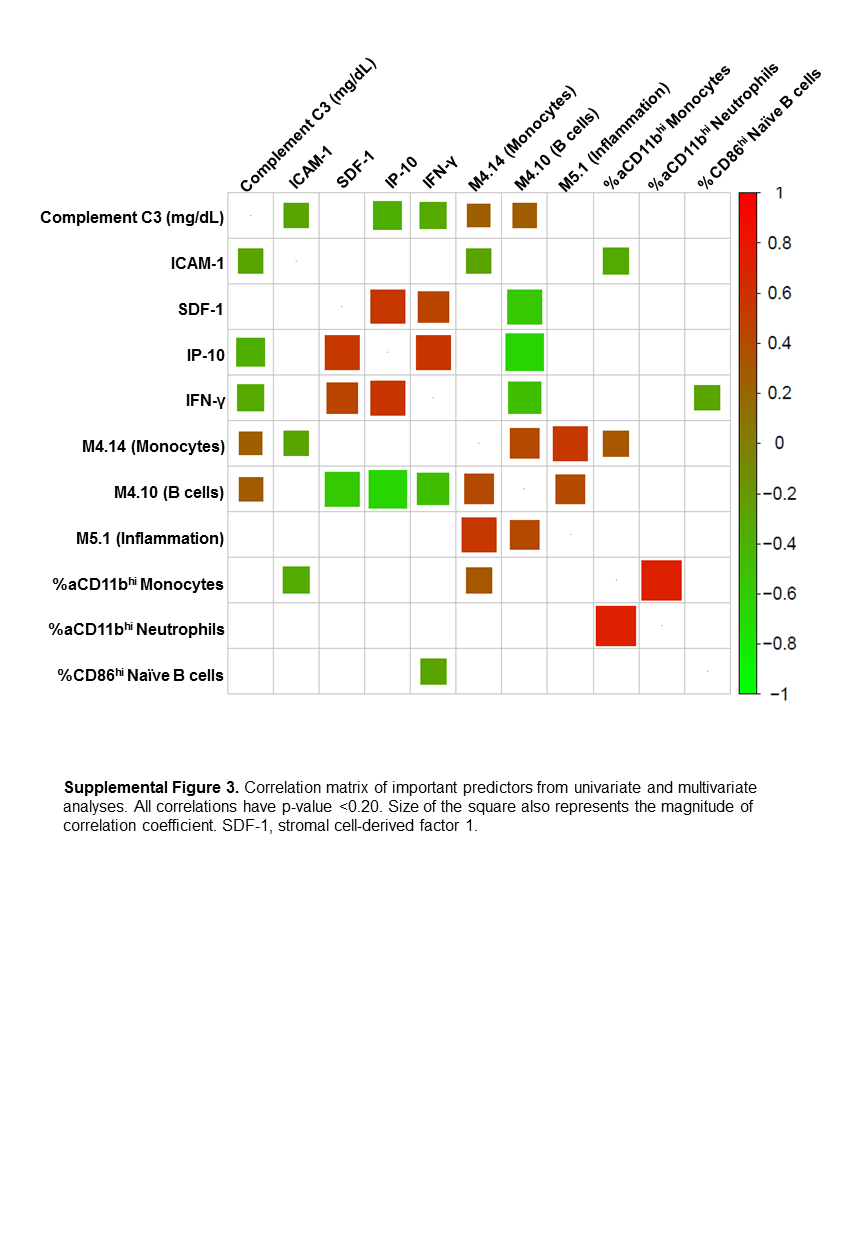
Supplementary Figure S2.** Correlation matrix of important predictors from univariate and multivariate analyses. All correlations have p-value <0.20. Size of the square also represents the magnitude of the correlation coefficient. SDF-1, stromal cell-derived factor 1.

**
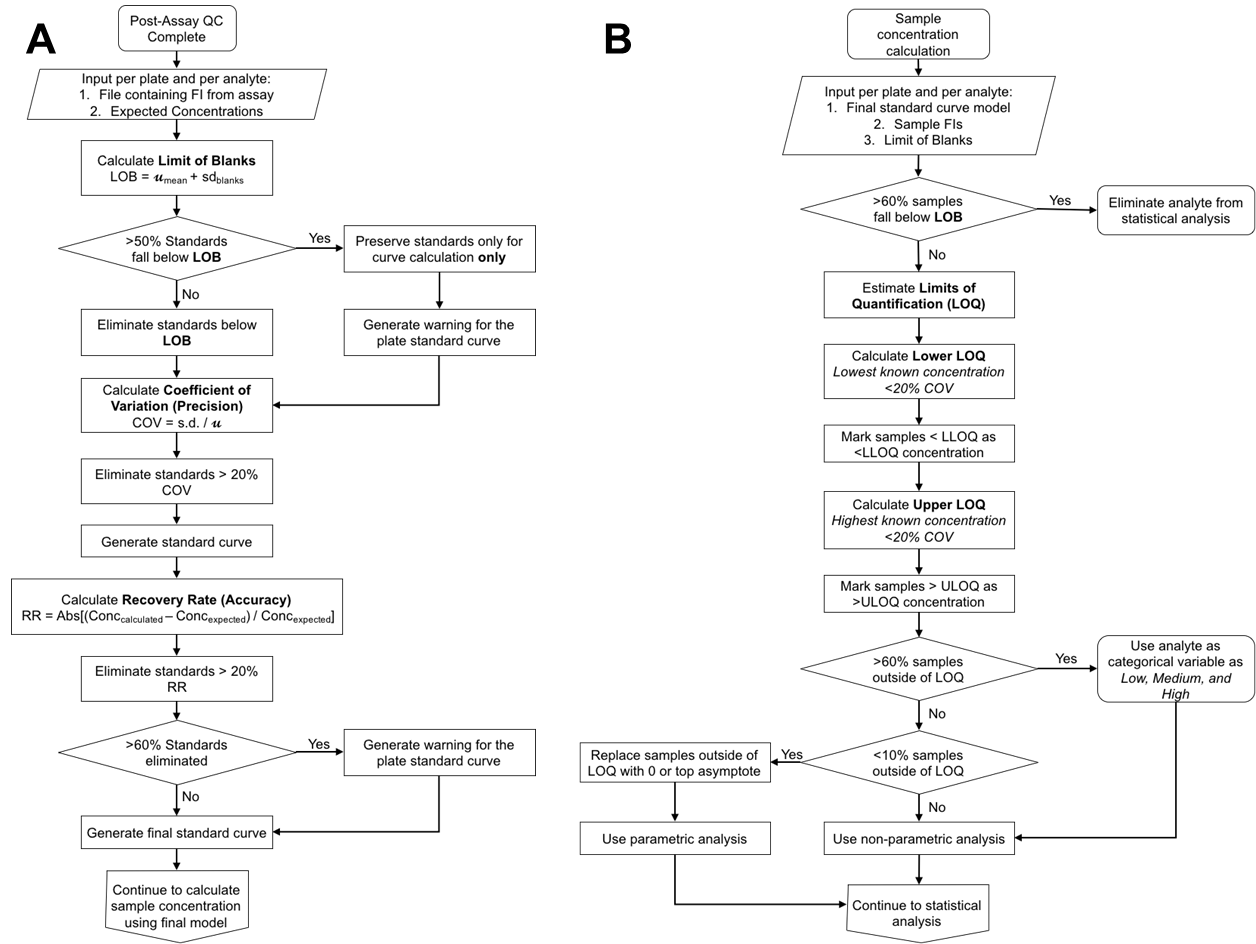
Supplementary Figure S3. Quality control algorithm used for soluble mediator data prior to statistical analysis.** (**A**) First step of the algorithm. Undetectable standards were eliminated based on limit of blanks (LOB). Unreliable standards were eliminated from the standard curve analysis based on coefficient of variation (COV) and recovery rate (RR). Remaining standards were then used to generate a sigmoidal curve based on 5-parameter logistic regression. (**B**) Second step of the algorithm. Undetectable samples were eliminated based on LOB. Samples between LOB and lower limit of quantification (LLOQ) were reported as “detectable but non-quantifiable” or LLOQ value of the corresponding analyte. Samples above upper limit of quantification (ULOQ) were reported as “high” or ULOQ value of the corresponding analyte.

**
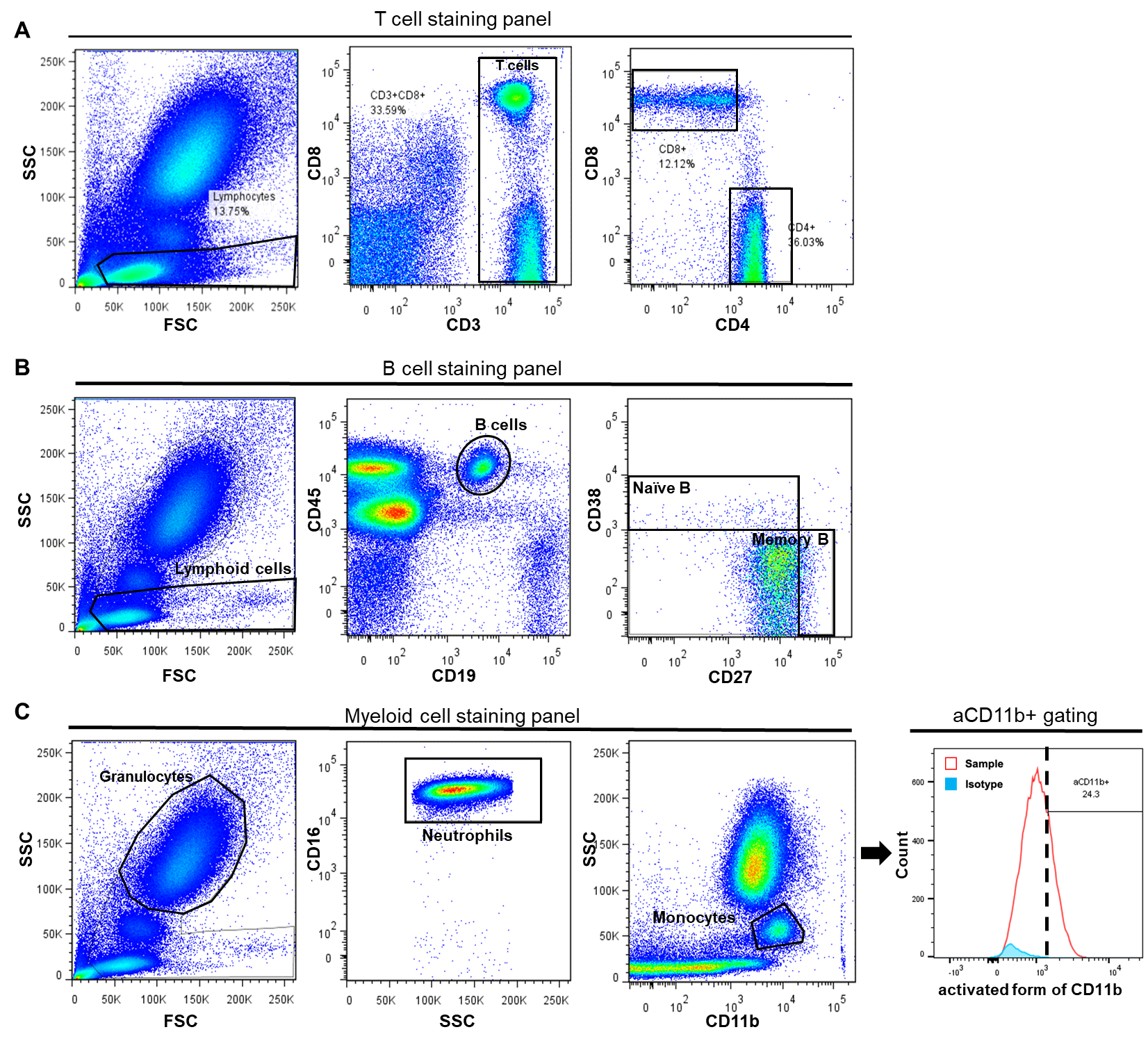
Supplementary Figure S4.** Flow cytometry gating strategies for T cells (**A**), B cells (**B**), and myeloid cells (**C**). Monocytes were also backgated to be CD38+. Activated neutrophils and monocytes were identified by positive aCD11b expression, with a positive cutoff determined using an isotype control

**Supplementary Table S1.** **Clinical panel values in SLE patients with early or late flare after steroid-induced disease suppression.**

|  | **Early flare,  Median (IQR)** | **Late flare,  Median (IQR)** | **p-value^1^** | **q-value^2^** |
| --- | --- | --- | --- | --- |
| Carbon Dioxide (mmol/L) | 24.5  (23.0 – 25.0) | 23.0  (22.0 – 24.0) | **0.01** | 0.37 |
| Complement C3 | 129.5  (122.2 – 156.8) | 117.5  (94.5 – 126.2) | **0.04** | 0.49 |
| % Monocytes | 7.10  (6.70 - 9.80) | 6.40  (5.10 - 7.70) | **0.06** | 0.86 |
| Chloride (mmol/L) | 105  (103 - 106) | 106  (104 - 107) | 0.12 | 0.86 |
| Urine WBC | 0.000  (0.000 - 13.3) | 0.000  (0.000 – 0.000) | 0.13 | 0.86 |
| Hematocrit (%) | 39.1  (35.0 - 40.8) | 40.1  (38.0 - 41.5) | 0.15 | 0.86 |
| Liver Function Test AST (U/L) | 19.0  (14.0 - 25.8) | 22.0  (17.0 – 34.0) | 0.17 | 0.86 |
| HDL (mg/dL) | 54.0  (46.3 - 69.8) | 50.0  (37.5 - 60.5) | 0.19 | 0.86 |
| Urine specific gravity | 1.02  (1.01 - 1.02) | 1.01  (1.01 - 1.02) | 0.19 | 0.86 |
| Potassium (mmol/L) | 4.10  (3.83 - 4.30) | 4.30  (3.90 - 4.45) | 0.23 | 0.86 |
| Albumin (g/dL) | 4.40  (4.30 - 4.70) | 4.20  (4.00 - 4.65) | 0.24 | 0.86 |
| Urine pH | 6.00  (5.63 - 6.88) | 6.50  (6.00 - 6.88) | 0.30 | 0.86 |
| Absolute basophil count (cells/uL) | 22.0  (16.75 - 30.5) | 28.0  (18.0 – 47.0) | 0.30 | 0.86 |
| MCHC (g/dL) | 33.5  (33.0 – 33.8) | 33.2  (32.5 - 33.8) | 0.30 | 0.86 |
| Liver function test, ALT (U/L) | 19.0  (11.0 – 26.0) | 21.0  (14.5 - 39.5) | 0.30 | 0.86 |
| Alkaline phosphate (U/L) | 66.5  (58.25 - 77.8) | 77.0  (59.5 - 90.5) | 0.30 | 0.86 |
| RBC count (million cells/uL) | 4.14  (3.97 - 4.48) | 4.30  (4.07 - 4.51) | 0.30 | 0.86 |
| Platelet count (thousands/uL) | 223  (189 - 297) | 257  (246 - 310) | 0.32 | 0.86 |
| Creatinine (mg/dL) | 0.805  (0.725 - 0.945) | 0.740  (0.635 - 0.910) | 0.35 | 0.86 |
| Absolute eosinophils (cells/uL) | 55.5  (31.3 - 102) | 112  (13.0 - 213) | 0.36 | 0.86 |
| Total ACR score | 5.00  (5.00 – 6.00) | 6.00  (5.00 – 7.00) | 0.36 | 0.86 |
| WBC (thousands/uL) | 5.35  (3.85 - 7.48) | 6.40  (4.45 - 7.95) | 0.39 | 0.86 |
| Cholesterol-to-HDL ratio | 3.30  (2.90 - 4.08) | 3.90  (2.85 - 4.70) | 0.40 | 0.86 |
| Sodium (mmol/L) | 140  (139 - 142) | 140  (139 – 141) | 0.41 | 0.86 |
| Absolute lymphocytes (1000x cells/uL) | 1.43  (0.989 – 1.82) | 1.67  (1.02 – 2.37) | 0.41 | 0.86 |
| Hemoglobin (g/dL) | 13.3  (11.4 - 13.7) | 13.2  (12.7 - 13.8) | 0.43 | 0.86 |
| MCV (fL) | 89.6  (84.5 - 98.9) | 93.4  (91.8 - 95.4) | 0.43 | 0.86 |
| Cholesterol (mg/dL) | 183  (157 - 223) | 174  (151 - 217) | 0.46 | 0.86 |
| Triglycerides (mg/dL) | 149  (115 - 179) | 152  (108 - 244) | 0.46 | 0.86 |
| Absolute neutrophils  (1000x cells/uL) | 3.64  (1.87 – 4.95) | 3.90  (2.05 – 5.80) | 0.47 | 0.86 |
| Urine leukocyte esterase | 1.00  (0.25 – 1.00) | 1.00  (0.00 – 1.00) | 0.47 | 0.86 |
| Urine nitrite | 0.00  (0.00 – 0.00) | 0.00  (0.00 – 0.00) | 0.48 | 0.86 |
| Calcium (mg/dL) | 9.40  (9.13 - 9.75) | 9.30  (8.80 - 9.55) | 0.48 | 0.86 |
| Urine bacteria | 0.00  (0.00 – 3.00) | 0.00  (0.00 – 0.00) | 0.52 | 0.89 |
| % basophils | 0.40  (0.300 - 0.700) | 0.400  (0.300 - 0.600) | 0.54 | 0.89 |
| % neutrophils | 59.4  (51.4 - 71.1) | 62.0  (47.1 - 72.5) | 0.58 | 0.90 |
| Albumin-to-globulin ratio | 1.50  (1.33 - 1.60) | 1.40  (1.00 - 1.75) | 0.60 | 0.91 |
| LDL (mg/dL) | 101  (79.5 - 122) | 84.0  (68.5 - 135) | 0.62 | 0.91 |
| Urine glucose | 0.500  (0.000 – 1.00) | 1.00  (0.000 – 1.00) | 0.67 | 0.92 |
| Urine bilirubin | 0.500  (0.000 – 1.00) | 1.00  (0.000 – 1.00) | 0.67 | 0.92 |
| MCH (pg) | 30.2  (28.0 - 33.0) | 31.0  (29.8 - 32.1) | 0.67 | 0.92 |
| Bilirubin (mg/dL) | 0.300  (0.300 - 0.475) | 0.300  (0.300 - 0.500) | 0.71 | 0.93 |
| Urine protein | 1.00  (0.000 – 1.00) | 1.00  (0.000 – 1.00) | 0.75 | 0.93 |
| Urine ketones | 0.500  (0.000 – 1.00) | 1.00  (0.000 – 1.00) | 0.79 | 0.93 |
| BUN (mg/dL) | 12.0  (9.25 - 14.8) | 12.0  (10.5 – 14.0) | 0.80 | 0.93 |
| Urine occult blood | 1.00  (0.000 – 1.00) | 1.00  (0.000 – 1.00) | 0.80 | 0.93 |
| % lymphocytes | 28.5  (20.9 - 37.3) | 28.7  (20.9 - 39.2) | 0.80 | 0.93 |
| % eosinophils | 1.20  (0.600 - 1.90) | 1.50  (0.500 - 2.90) | 0.83 | 0.93 |
| Urine squamous epithelial cells | 0.000  (0.000 - 4.50) | 0.000  (0.000 - 4.50) | 0.84 | 0.93 |
| Urine RBC | 0.000  (0.000 – 0.000) | 0.000  (0.000 – 0.000) | 0.85 | 0.93 |
| Glucose (mg/dL) | 87.5  (77.8 – 91.0) | 82.0  (78.5 - 90.5) | 0.88 | 0.95 |
| Total protein (g/dL) | 7.30  (7.10 - 7.78) | 7.40  (7.00 – 8.00) | 0.94 | 0.98 |
| Absolute monocytes (cells/uL) | 418  (322 - 491) | 408  (301 - 521) | 0.95 | 0.98 |
| Globulin (g/dL) | 3.00  (2.63 - 3.28) | 3.00  (2.60 - 3.70) | 0.97 | 0.99 |
| RDW (%) | 14.6  (13.8 - 16.1) | 14.4  (13.8 - 16.6) | 0.99 | 0.99 |

^1^by Wilcoxon rank-sum test. Bold p-values indicate variables included in multivariable modeling (threshold of p<0.10). ^2^Adjusted p-values based on false discovery rate.

**Supplementary Table S2.** **Autoantibody positivity and levels in SLE patients with early or late flare after steroid-induced disease suppression, by multiplex bead-based assay.**

| **Autoantibodies** | **Early flare,  Median (IQR)** | **Late flare,  Median (IQR)** | **p-value^1^** | **q-value^2^** | **Early flare,  No. Positive (%)** | **Late flare,  No. Positive (%)** | **p-value^3^** | **q-value^2^** |
| --- | --- | --- | --- | --- | --- | --- | --- | --- |
| dsDNA | 2 (1 - 8.34) | 4 (1 - 7) | 0.59 | 0.944 | 5 (23.81%) | 2 (15.38%) | 0.68 | 1 |
| Chromatin | 0.2 (0.18 - 0.82) | 0.2 (0.15 - 2.2) | 0.65 | 0.944 | 5 (23.81%) | 4 (30.77%) | 0.70 | 1 |
| Ribosomal P | 0.2 (0.08 - 0.2) | 0.2 (0.1 - 0.2) | 0.76 | 0.944 | 2 (9.52%) | 1 (7.69%) | 1.00 | 1 |
| Ro/SSA | 0.2 (0.2 - 1.1) | 0.3 (0.2 - 6.05) | 0.23 | 0.749 | 1 (4.76%) | 2 (15.38%) | 0.54 | 1 |
| 52 kD Ro/SSA | 0.2 (0.01 - 0.2) | 0.2 (0.1 - 0.5) | 0.44 | 0.818 | 2 (9.52%) | 2 (15.38%) | 0.63 | 1 |
| 60 kD Ro/SSA | 0.2 (0.2 - 0.4) | 0.2 (0.15 - 4.4) | 0.47 | 0.818 | 3 (14.29%) | 4 (30.77%) | 0.39 | 1 |
| La/SSB | 0.2 (0.05 - 7.8) | 0.2 (0 - 7.19) | 0.96 | 0.956 | 6 (28.57%) | 4 (30.77%) | 1.00 | 1 |
| Sm | 0.2 (0.08 - 0.2) | 0.2 (0.2 - 1.54) | 0.17 | 0.749 | 4 (19.05%) | 3 (23.08%) | 1.00 | 1 |
| SmRNP | 0.2 (0.2 - 1.69) | 0.2 (0.2 - 4.7) | 0.90 | 0.956 | 6 (28.57%) | 4 (30.77%) | 1.00 | 1 |
| RNP | 0.2 (0.18 - 8.1) | 8.1 (0.6 - 8.1) | 0.08 | 0.749 | 1 (4.76%) | 1 (7.69%) | 1.00 | 1 |
| RNP A | 0.2 (0.19 - 8.1) | 1 (0.2 - 8.1) | 0.40 | 0.818 | 8 (38.1%) | 8 (61.54%) | 0.29 | 1 |
| RNP68 | 0.2 (0.01 - 0.2) | 0.2 (0 - 0.2) | 0.78 | 0.944 | 2 (9.52%) | 1 (7.69%) | 1.00 | 1 |
| Centromere B | 0.2 (0.01 - 0.2) | 0.2 (0 - 0.2) | 0.24 | 0.749 | 2 (9.52%) | 0 (0%) | 0.51 | 1 |
| Scl 70 | 0.2 (0.08 - 0.3) | 0.2 (0 - 0.2) | 0.25 | 0.749 | 1 (4.76%) | 1 (7.69%) | 1.00 | 1 |
| Jo1 | 0.2 (0 - 0.2) | 0.2 (0 - 0.2) | 0.95 | 0.956 | NA | NA | N/A | NA |
| These assays were performed at entry to the study: all patients had a previous history of a positive ANA. Anti-dsDNA was quantified in IU/mL with a positive cutoff of 10 IU/mL; all others were quantified using an antibody index value, with a positive cutoff of AI=1. ^1^p-values calculated by Wilcoxon rank-sum test. Bold p-values indicate variables included in multivariable modeling (threshold of p<0.10); ^2^q-values adjusted by false discovery rates; ^3^p-values calculated by Chi-square test | | | | | | | | |

**Supplementary Table S3.** **Baseline soluble mediator values in SLE patients with early or late flare after steroid-induced disease suppression.**

| **Mediator** | **Early Flare, pg/mL^1^ Median (IQR)** | **Late Flare, pg/mL Median (IQR)** | **p-value^2^** | **q-value^3^** |
| --- | --- | --- | --- | --- |
| IFN-γ | 0.27 (0.23 - 0.40) | 0.52 (0.34 - 1.35) | **0.02** | 0.66 |
| TNFRII | 158 (129 - 195) | 195 (163 - 292) | **0.07** | 0.73 |
| TNFRI | 53.0 (48.0 - 71.0) | 79.1 (54.2 - 117.23) | **0.08** | 0.73 |
| ICAM-1 | 173081 (86095 - 241395) | 241395 (170817 - 241395) | 0.12 | 0.73 |
| GROα | 9.06 (3.91 - 16.0) | 12.7 (6.28 - 26.7) | 0.15 | 0.73 |
| Resistin | 1529 (1228 - 2035) | 1738 (1507 - 2053) | 0.16 | 0.73 |
| Leptin | 16037 (8248 - 27403) | 8801 (5252 - 21201) | 0.18 | 0.73 |
| PAI-1 | 843 (318 - 2053) | 1060 (522 - 2813) | 0.20 | 0.73 |
| IL-2Rα | 18.2 (14.8 - 31.8) | 26.4 (18.2 – 66.0) | 0.23 | 0.76 |
| Eotaxin | 67.5 (34.8 - 117.0) | 96.7 (43.8 - 151) | 0.30 | 0.90 |
| SDF-1 | 1565 (1371 - 2037) | 1715 (1560 - 1837) | 0.35 | 0.91 |
| MCP-1 | 39.0 (24.6 - 67.2) | 41.6 (30.9 - 98.8) | 0.42 | 0.91 |
| APRIL | 2010 (0.00 - 6087) | 0.00 (0.00 - 2388) | 0.42 | 0.91 |
| TRAIL | 31.9 (22.7 - 44.9) | 35.7 (22.2 - 68.3) | 0.43 | 0.91 |
| MCP-3 | 95.3 (73.9 - 132) | 109 (80.2 - 166) | 0.44 | 0.91 |
| SCF | 9.63 (8.57 - 15.9) | 11.7 (9.01 - 14.6) | 0.50 | 0.94 |
| IL-17A | 0.82 (0.82 - 0.82) | 0.82 (0.82 - 0.85) | 0.51 | 0.94 |
| LIF | 1.52 (1.02 - 1.78) | 1.72 (0.79 - 2.81) | 0.54 | 0.94 |
| MIG | 50.4 (38.4 - 89.6) | 39.0 (33.4 - 203) | 0.58 | 0.96 |
| BLyS | 943 (836 – 1370) | 1247 (795 - 1585) | 0.67 | 0.99 |
| IL-23 | 0.61 (0.36 - 1.01) | 0.36 (0.36 - 1.02) | 0.68 | 0.99 |
| RANTES | 145 (94.6 - 203) | 145 (109 - 297) | 0.71 | 0.99 |
| sCD40L | 35.6 (13.6 - 83.2) | 33.2 (16.7 - 314.4) | 0.76 | 0.99 |
| MIP-1β | 102 (66.2 - 164) | 82.7 (49.2 - 210) | 0.78 | 0.99 |
| MIP-1α | 13.0 (7.73 - 45.5) | 14.8 (6.00 - 54.5) | 0.82 | 0.99 |
| sVCAM | 8600. (746 - 10491) | 7927 (6432 - 10569) | 0.82 | 0.99 |
| VEGF-A | 29.6 (11.6 - 220.8) | 69.9 (9.70 - 113) | 0.84 | 0.99 |
| PDGF-BB | 5.40 (3.76 - 10.6) | 4.97 (3.89 – 16.0) | 0.91 | 1.00 |
| IP-10 | 35.62 (17.58 – 85.00) | 27.98 (18.93 - 94.14) | 0.92 | 1.00 |
| sE-selectin | 2250 (1542 - 3191) | 2167 (1792 - 36634) | 0.95 | 1.00 |
| IL-1α | 8.64 (1.28 – 189.0) | 5.12 (1.38 - 17.1) | 1.00 | 1.00 |
| IL-21 | 1.60 (0.78 - 2.04) | 1.18 (0.76 - 2.21) | 1.00 | 1.00 |

^1^Plasma concentration. ^2^Calculated by Wilcoxon rank-sum test. Bold p-values indicate variables included in multivariable modeling (threshold of p<0.10). ^3^Adjusted p-values based on false discovery rate.

**Supplementary Table S4. Flow cytometry profiling panels used for analysis of cell types and states**.

| **Panel** | **Antibody** | **Manufacturer** | **Catalog Number** |
| --- | --- | --- | --- |
| **T cell** | anti-CD3-PE-Cy7 | Biolegend | 300420 |
|  | anti-CD279-AF647 | Biolegend | 329910 |
|  | anti-CD194-PerCP-Cy5.5 | BD Pharmingen | 560726 |
|  | anti-CD80-PE-Cy5 | BD Pharmingen | 560442 |
|  | anti-CD4-AlexafluorAF)700 | Biolegend | 317426 |
|  | anti-CD8-AF610-PE | Invitrogen | MHCD0822 |
|  | anti-CD275-FITC | AbDSerotec | MCA2629F |
|  | anti-CD154-AF750 | eBiosciences | 555701 |
|  | and anti-IL-21R-PE | BD Biosciences | 560264 |
|  |  |  |  |
| **B cell** | anti-CD45-APC/Cy7 | Biolegend | 304014 |
|  | anti-CD27-AF700 | BD Pharmingen | 560611 |
|  | anti-CD40-FITC | Biolegend | 303604 |
|  | anti-CD194-PerCP-Cy5.5 | BD Pharmingen | 560726 |
|  | anti-CD80-PE-Cy5 | Biolegend | 305210 |
|  | anti-CD86-V450 | BD Pharmingen | 560359 |
|  | anti-CD19-PE-Texa Red | Abcam | AB51386 |
|  | and anti-IL21R-PE | BD Biosciences | 560264 |
|  |  |  |  |
| **Myeloid cell** | anti-CD45-PE-Texas Red | Invitrogen | MHCD4517 |
|  | anti-CD209-PerCP-Cy5.5 | Biolegend | 330110 |
|  | anti-CD3/CD4-AF700 | Biolegend | 317426 |
|  | anti-CD56-PE-Cy7 | BioLegend | 560361 |
|  | anti-CD11b-PE-Cy5 | BioLegend | 301308 |
|  | anti-CD11b active form)-FITC | BioLegend | 301404 |
|  | anti-IL21R-PE | BD Biosciences | 560264 |
| Anti-activated CD11b (CBRM1/5) antibody specifically recognizes the epitope exposed after activation-induced conformational change. | | | |

**Supplementary Table S5.** **Cellular panel measured by flow cytometry in SLE patients with early or late flare after steroid-induced disease suppression.**

| **Parameter** | **Early flare,  Median (IQR)** | **Late flare,  Median (IQR)** | **p-value^1^** | **q-value^2^** |
| --- | --- | --- | --- | --- |
| %aCD11b+ Neutrophil | 2.41 (0.03 - 11.98) | 0.01 (0 - 0.04) | **0.00** | 0.07 |
| %aCD11b+ Monocytes | 1.14 (0.03 - 5.64) | 0.03 (0 - 0.05) | **0.03** | 0.23 |
| %CD86^hi^ Naïve B | 3.31 (0.52 - 8.10) | 0.65 (0.33 - 1.40) | **0.03** | 0.23 |
| Live B cells count | 207817  (147568-250804) | 292795  (205010 - 391755) | **0.07** | 0.38 |
| CD40 on Naïve B, Geo Mean FI | 1560 (438 - 2356) | 674 (0 - 1654) | 0.10 | 0.38 |
| %Naive B cells | 45.1 (26.0 - 75.2) | 78.9 (46.3 – 91.0) | 0.10 | 0.38 |
| CD11b on Monocytes, Geo Mean FI | 12269  (7085 - 27400) | 17900  (12000 - 39700) | 0.13 | 0.40 |
| Live Monocytes count | 214465  (134161 - 243543) | 243394  (186886 - 386000) | 0.16 | 0.43 |
| CD11b on Neutrophils, Geo Mean FI | 10292  (6346 - 16493) | 17417  (8812 - 28476) | 0.19 | 0.47 |
| Total cell count | 46512  (14800 - 86372) | 77884  (33475 - 106975) | 0.31 | 0.65 |
| Live Neutrophils count | 214465  (134161 - 243543) | 235142  (174222 - 374451) | 0.33 | 0.65 |
| Naïve B count | 1951 (572 - 4706) | 3045 (958 - 7764) | 0.41 | 0.76 |
| CD11b Monocyte count | 7853  (4715 - 13665) | 10083  (7938 - 13702) | 0.50 | 0.78 |
| %Naive B cells | 1.91 (1.18 - 5.72) | 2.45 (0.62 - 3.93) | 0.60 | 0.78 |
| IL.21r on Naïve B, Geo Mean FI | 1951 (1272 - 2540) | 1349 (1081-3091) | 0.60 | 0.78 |
| %IL-21r^hi^ Monocyte | 5.10 (0.73 - 10.4) | 2.94 (0.03 - 16.97) | 0.61 | 0.78 |
| %CD11b^+^ Monocyte | 4.76 (3.09 - 5.62) | 4.14 (2.26 - 5.44) | 0.64 | 0.78 |
| %CD80^hi^ Naïve B | 0.85 (0.26 - 2.42) | 0.60 (0.19 - 2.24) | 0.71 | 0.82 |
| %Neutrophils | 32.9 (11.27 – 50.0) | 31.3 (7.34 - 46.9) | 0.95 | 1.00 |
| %IL-21r^hi^ Neutrophil | 3.36 (1.49 - 6.86) | 3.37 (0.02 - 14.6) | 0.97 | 1.00 |
| %CD194^hi^ Naïve B | 3.36 (0.80 - 8.42) | 3.36 (0.11 - 17.6) | 1.00 | 1.00 |
| ^1^Calculated by Wilcoxon rank-sum test. Bold p-values indicate variables included in multivariable modeling (threshold of p<0.10). ^2^ Adjusted p-values based on false discovery rate | | | | |

**Supplementary Table S6. Clinical manifestations in three subgroups of SLE patients with early or late flare after steroid-induced disease suppression.**

|  | Early Flare Group 1 (n = 5) | Early Flare Group 2 (n = 6) | Early Flare Group 3 (n = 5) | p-value |
| --- | --- | --- | --- | --- |
| Calculated scores  Median (IQR) |  |  |  |  |
| BILAG Score | 6.0 (10.8 - 19.3) | 5.0 (16 - 17) | 5.0 (16 - 16) | 0.640 |
| SELENA-SLEDAI Score | 6.0 (6.5 - 10.3) | 5.0 (8.0 - 9.0) | 8.0 (8.0 - 9.0) | 0.669 |
| ACR Total Score | 5.5 (5.0 - 7.5) | 5.0 (5.0 - 6.0) | 5.0 (5.0 - 6.0) | 0.812 |
|  |  |  |  |  |
| ACR Criteria  N (%) |  |  |  |  |
| Malar Rash | 2 (40%) | 3 (50%) | 3 (60%) | 0.819 |
| Discoid Rash | 3 (60%) | 2 (33%) | 1 (20%) | 0.411 |
| Photosensitivity | 4 (80%) | 3 (50%) | 4 (80%) | 0.456 |
| Oral Ulcers | 3 (60%) | 5 (83%) | 4 (80%) | 0.641 |
| Arthritis | 5 (100%) | 6 (100%) | 5 (100%) | N/A |
| Serositis | 2 (40%) | 3 (50%) | 3 (60%) | 0.819 |
| Renal Disorders | 1 (20%) | 1 (17%) | 0 (0%) | 0.587 |
| Neurological Disorders | 0 (0%) | 1 (17%) | 0 (0%) | 0.411 |
| Hematological Disorders | 1 (20%) | 1 (17%) | 0 (0%) | 0.587 |
| Immunological Disorders | 2 (40%) | 5 (83%) | 3 (60%) | 0.332 |
|  |  |  |  |  |
| SLEDAI Component  Median (IQR) |  |  |  |  |
| General | 1.0 (1.0 - 1.0) | 1.0 (1.0 - 1.0) | 1.0 (1.0 - 1.0) | 0.357 |
| Musculoskeletal Disorders | 5.0 (5.0 - 5.0) | 5.0 (5.0 - 5.0) | 8.5 (5.0 - 12) | 0.043 |
| Mucocutaneous | 5.0 (5.0 - 5.0) | 5.0 (5.0 - 5.0) | 1.0 (1.0 - 4.0) | 0.091 |
| Hematological Disorders | 0 (0 - 0) | 0 (0 - 1) | 0 (0 -1) | 0.418 |
| Renal Disorders | 0 (0 - 0) | 0 (0 - 0) | 0 (0 - 0) | 0.465 |
| Neurological Disorders | 0 (0 - 0) | 0 (0 - 0) | 0 (0 - 0) | N/A |
| Vasculitis | 1.0 (0 - 1.0) | 1.0 (1.0 - 1.0) | 1.0 (0.3 - 1.0) | 0.819 |
| Cardiovascular Disorders | 0 (0 - 0) | 0 (0 - 0) | 0 (0 - 0) | N/A |

Significant p-values are highlighted in bold; p-values are calculated with Wilcoxon non-parametric test and Fischer exact test

**Supplementary References**

1 Merrill, J. T. *et al.* The Biomarkers of Lupus Disease Study: A Bold Approach May Mitigate Interference of Background Immunosuppressants in Clinical Trials. *Arthritis & rheumatology (Hoboken, N.J.)* **69**, 1257-1266, doi:10.1002/art.40086 (2017).

2 Hochberg, M. C. Updating the American College of Rheumatology revised criteria for the classification of systemic lupus erythematosus. *Arthritis and rheumatism* **40**, 1725 (1997).

3 Tan, E. M. *et al.* The 1982 revised criteria for the classification of systemic lupus erythematosus. *Arthritis and rheumatism* **25**, 1271-1277 (1982).

4 Isenberg, D. A. *et al.* BILAG 2004. Development and initial validation of an updated version of the British Isles Lupus Assessment Group's disease activity index for patients with systemic lupus erythematosus. *Rheumatology* **44**, 902-906, doi:10.1093/rheumatology/keh624 (2005).

5 Yee, C. S. *et al.* The BILAG-2004 index is sensitive to change for assessment of SLE disease activity. *Rheumatology* **48**, 691-695, doi:10.1093/rheumatology/kep064 (2009).

6 Furie, R. A. *et al.* Novel evidence-based systemic lupus erythematosus responder index. *Arthritis and rheumatism* **61**, 1143-1151, doi:10.1002/art.24698 (2009).
